# Supplementary material for: Beneficial Effect of Methanolic Extract of Frankincense (Boswellia Sacra) on Testis Mediated through Suppression of Oxidative Stress and Apoptosis
Source: Molecules. 2022 Jul 22;27(15):4699. doi: 10.3390/molecules27154699 (PMC9332498; doi:10.3390/molecules27154699)
Supplement: Supplementary file 1 [file molecules-27-04699-s001.zip › molecules-1804249-supplementary.pdf]

molecules

MDPI

Supplementary Materials

Beneficial Effect of Methanolic Extract of Frankincense (*Boswellia Sacra*) on Testis Mediated through Suppression of Oxidative Stress and Apoptosis

Samir Abdulkarim Alharbi, Mohammed Asad*, Kamal Eldin Ahmed Abdelsalam, Monjid Ahmed Ibrahim and Sunil Chandy

Department of Clinical Laboratory Science, College of Applied Medical Sciences, Shaqra University, Shaqra P O Box 1383, Saudi Arabia; saalharbi@su.edu.sa (S.A.A.); kabdelsalam@su.edu.sa (K.E.A.A.); monjid@su.edu.sa (M.A.I.); schandy@su.edu.sa (S.C.)

* Correspondence: masad@su.edu.sa; Tel.: +966-50-4984-171

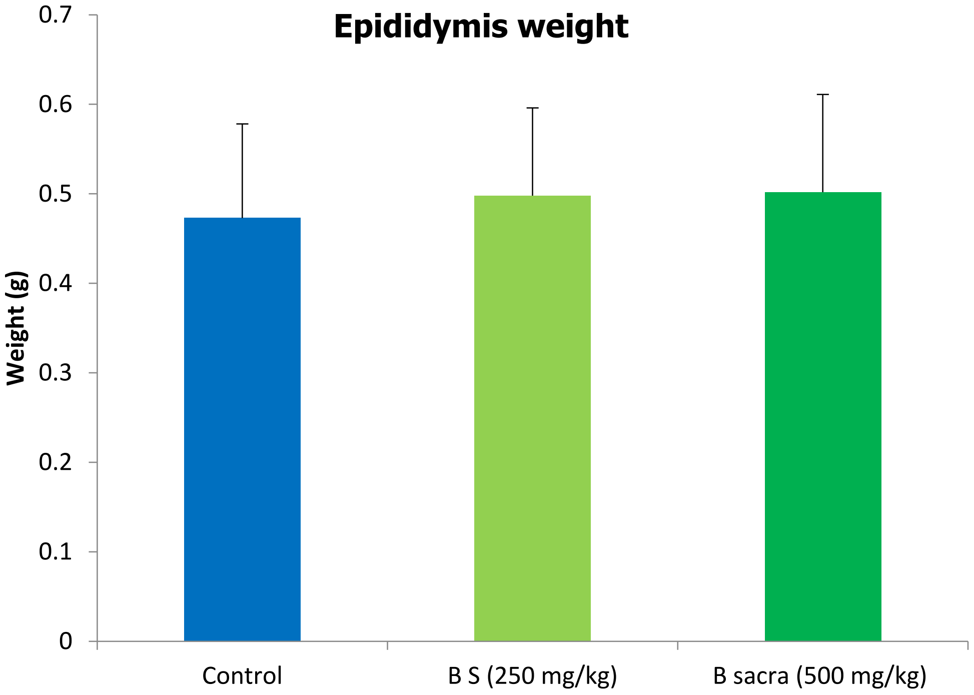

Figure S1. Effect on weight of epididymis after different treatments. All values are mean ± 6, *n* = 6, differences not significant from each other.

Table S1. List of constituents detected by GC-MS.

Number	Name of the Constituent	Rt	Area (%)
1.	2-Furanmethanol	3.526	0.79
2.	1,2-ethanediol diacetate	3.615	1.18
3.	Borane, Diethylmethyl	3.815	0.52
4.	4-Cyclopentene-1,3-dione	3.869	1.30
5.	Oxiranemethanol	4.035	0.48
6.	Acetopropanol	4.097	0.63
7.	Crotonyl isothiocyanate	4.176	1.05
8.	2(3H)-Furanone, Dihydro-4-hydroxy	4.286	0.94
9.	Alpha, beta Crotonolacton	4.330	0.86
10.	3(5) D1-1,2,4-triazole-d1	4.446	1.48
11.	Acetic acid, propyl ester	4.529	2.30
12.	Acetic acid, propyl ester	4.822	0.40
13.	1,2-cyclooctanedione	4.967	0.81
14.	2-Furanmethanol, 5-methyl-	5.076	0.41
15.	2-Furancarboxaldehyde, 5-methyl	5.223	0.74
16.	2,4-Dihydroxy-2,5-dimethyl-3(2H)-furanone	5.508	0.65
17.	Ethanol, 2-[(triethylsilyl)oxy]-	5.598	0.40
18.	Phenol	5.696	0.76
19.	2-Hydroxy-gamma-butyrolactone	5.861	2.20
20.	2H-Pyran-2-one, tetrahydro-3,6-dimethyl	6.091	1.00
21.	7-Oxa-bicyclo[2.2.1]hept-5-en-2-one	6.376	0.99
22.	2-Cyclopenten-1-one, 2-hydroxy	6.599	0.90
23.	3-Methylpent-2-ene-1,5-diol	6.776	0.84
24.	Proceroside	6.883	2.15
25.	1,4-Dioxin, 2,3-dihydro-5,6-dimethyl	7.069	2.06
26.	2,5-Anhydro-1,6-dideoxyhexo-3,4-diulose	7.548	1.73
27.	2-Pyrrolidinone	7.867	0.42
28.	2,3-Dihydro-5-hydroxy-6-methyl-4(H)-pyran-4-one	7.961	1.71
29.	Pentanal	8.152	1.16
30.	2-Butene, 1,4-diethoxy-	9.065	0.38
31.	2-Acetyl-2-hydroxy-.gamma.- butyrolactone	9.285	0.54
32.	2,3-Dihydro-3,5-dihydroxy-6-methyl-4H-pyran-2-one	9.544	3.41
33.	1,1,3,3-Tetramethyl-1,3-bis[3-(2-oxiranylmethoxy)propyl]disiloxane	9.726	0.55
34.	Silane, [(1,1-dimethyl-2-propenyl)oxy]dimethyl	10.107	0.60
35.	1,2-Dioxetane, 3,4,4-trimethyl-3-[[(trimethylsilyl)oxy]methyl]-	10.258	0.39
36.	Trimethyltetrahydropyran	10.520	0.73
37.	1,2-Benzenediol	11.322	0.65
38.	Benzofuran, 2,3-dihydro-	11.608	0.98
39.	5-Hydroxymethylfurfural	11.856	2.25
40.	2-methoxy-4-vinylphenol	13.932	0.35
41.	Formic acid, hex-2-yl ester	14.320	0.50
42.	3-Azetidin-1-yl-propionic acid, methyl ester	14.422	0.45
43.	3-phenyl-2-thioxopropanoic acid	14.470	0.50

44.	Methyl-6-deoxyhexapyranoside	15.188	0.75
45.	2- Furanmethanol, 5-ethenyl	15.854	0.39
46.	Benzaldehyde, 2-hydroxy-6-methyl-	17.567	0.55
47.	2(4H)-Benzofuranone, 5,6,7,7a-tetrahydro	19.230	2.03
48.	3-Ethoxy-4-hydroxyphenyl acetonitrile	23.743	9.57
49.	Tetradecanoic acid	24.109	0.74
50.	2(4H)-Benzofuranone, 5,6,7,7a-tetrahydro-6-hydroxy-4,4,7a-trimethyl-	24.187	1.82
51.	2-Pentadecanone, 6,10,14-trimethyl-	25.356	0.47
52.	Hexadecanoic acid, 2-hydroxy-1-(hydroxymethyl)ethyl ester	26.516	0.69
53.	Pentadecanoic acid	27.101	6.70
54.	5-Chloro-2,2-dimethylpentanenitrile	27.430	0.63
55.	Benzenemethanol, 2,5-dimethoxy-, acetate	27.566	1.46
56.	beta.-D-mannofuranoside, 1-O-(10-undecenyl)-	28.012	3.98
57.	Heptadecanoic acid	28.311	1.00
58.	9,12,15-Octadecatrienoic acid	28.757	0.63
59.	Phytol	28.912	1.50
60.	9,12,15-Octadecatrienoic acid, (Z,Z,Z)-	29.346	6.80
61.	Tricyclo[7.1.0.0[1,3]]decane-2-carbaldehyde	29.460	0.83
62.	Octadecanoic acid	29.595	2.13
63.	hahnfett	30.809	0.70
64.	Malonic acid, 2-butyl tetradecyl ester	31.898	0.85
65.	Icosanoic acid	32.015	0.52
66.	Ethyl 1-thio-.alpha.-l-arabinofuranoside	33.310	3.25
67.	Hexadecanoic acid, methyl ester	33.468	2.52
68.	.beta.-l-Rhamnofuranosid, 5-O-acetyl-tio-octyl-	33.771	0.41
69.	Phthalic acid, di(2-propylpentyl) ester	33.828	0.35
70.	.beta.-l-Rhamnofuranosid, 5-O-acetyl-tio-octyl-	34.026	0.73
71.	2,3-Dihydroxypropyl elaidate	35.076	0.78
72.	Ethyl (9z,12z)-9,12-octadecadienoate	35.123	1.85
73.	Octadecanoic acid, 2,3-dihydroxypropyl ester	35.275	1.15
74.	2,8-Dimethyl-2-(4,8,12-trimethyltridecyl)-6-chromanol	36.364	0.45
75.	2,8-Dimethyl-2-(4,8,12-trimethyltridecyl)-6-chromanol	36.526	0.64
